# Challenges and barriers to e-learning experienced by trainers and training coordinators in the Ministry of Health in Saudi Arabia during the COVID-19 crisis

**DOI:** 10.1371/journal.pone.0274816

**Published:** 2022-10-17

**Authors:** Dalil Al Shamari

**Affiliations:** Assistant Deputyship of Hospital Affairs—MOH HQ- Riyadh, Kingdom of Saudi Arabia; Unaizah College of Pharmacy, Qassim University, SAUDI ARABIA

## Abstract

**Introduction:**

The sudden shutdown caused by coronavirus disease 2019 has far-reaching effects, including on education and training. For this reason, traditional education and training have shifted to an online learning format. This study explores the challenges of and barriers to e-learning experienced by trainers and training coordinators in the Saudi Ministry of Health during the coronavirus disease 2019 pandemic.

**Methods:**

A cross sectional survey was distributed among participants by email. The sample included 262 trainers and training coordinators currently working for the Saudi Ministry of Health in hospitals, primary health care centers, and training centers (including general directorates of regions and clusters) in all 13 administrative regions of the country.

**Results:**

Most participants exhibited an intermediate level of experience with e-learning (58.4%) and found the task of adapting to unfamiliar technology to be a challenge (22.1%). Limited social interaction in relation to cheating/plagiarism contexts, frequent technological failures, and a lack of policies or standards for e-learning were significant barriers for participants; these were mentioned by 46.9%, 43.5%, and 40.1% of participants, respectively.

**Conclusions:**

This study highlights the challenges and barriers encountered in the adoption of e-learning by trainers and training coordinators in the Saudi Ministry of Health. The challenges of and barriers to e-learning included but were not limited to communication, assessment of trainees, adaptation to a lack of policy, and frequent technology failure. Adapting to new technologies is challenging for trainers and training coordinators, which is exacerbated by a lack of adequate policies and standards to eliminate cheating and avoid technological failures. These results could help bridge gaps in the use of e-learning by improving policies, holding workshops and training sessions, and providing continuous information technology support in e-learning.

## Introduction

The COVID-19 pandemic has had far-reaching effects, including on the economy and education [1, 2]. The World Health Organization (WHO) [1] called for social distancing as a way of curbing the spread of the COVID-19 pandemic. Social distancing has been defined by the Red Cross [3] as the conscious increase of physical distance between people to reduce the spread of an infectious disease. In March 2020, the Saudi government issued stay-at-home orders, and the Ministry of Education announced the closure of schools and universities as recommended by the Ministry of Health (MOH) authorities to protect students and staff [4]. Following the closure of institutions and training centers due to COVID-19 concerns, education changed dramatically, including the notable rise of electronic learning (e-learning), which is conducted remotely via digital platforms [5]. Thus, e-learning is the most appropriate way to present an educational curriculum and to conduct assessment and examination. The COVID-19 pandemic may come to be seen as a significant turning point in the use of e-learning across the world [6].

The term e-learning refers to more than online learning, virtual learning, and networked or web-based learning. Here, “electronic,” designated by the letter “e” in the term e-learning, takes into account all educational activities in which individuals or groups, working online or offline, participate, synchronously or asynchronously, via networks, computers, or any other type of electronic devices [7, 8]. Thus, e-learning is a vital element in enabling the continuation of education, and the authorities have ably enhanced its capabilities in response to the pandemic. However, virtual learning cannot substitute for face-to-face learning and in-person classes [9].

E-learning provides learners with easier and more effective access to a variety of information. However, the transition from traditional education to e-learning is not without challenges. Learners and educators alike are working under unusual time limitations and in the face of enhanced demand, which is driving institutions to seek new means of providing a personalized, self-directed learning experience [10].

Moreover, the worldwide experience and acceptance of contemporary e-learning caused trainers, training coordinators, and learners to become accustomed to using technological devices and tools for training and learning [11, 12]. The job of a trainer is to present information and direct structured learning experiences to increase the skills and/or knowledge of the trainees. Trainers also design and develop training programs, conduct pre- and post-tests, prepare education materials, plan for appropriate education locations, including laboratories and classrooms, and ensure trainee engagement [13]. In the context of the Saudi MOH, trainers are either clinical or nonclinical trainers, and training coordinators are responsible for administrative support for training functions, including creating an annual training schedule for an institute after assessing its needs, ordering packaged training materials, preparing a suitable location for the training, maintaining audiovisual equipment, registering the trainees, scheduling the training sessions, and summarizing the course evaluation [13].

Nonetheless, several issues occurred during the pandemic period in relation to e-learning. These included certain intrinsic and extrinsic factors that could have influenced the perceptions, involvement, and participation of the trainers/training coordinators and trainees in online instruction [14]. For instance, the age of trainers and training coordinators may affect their ability to provide e-learning due to differences in experience with technology across generations. An American study found that older workers tend to have lower level of digital skills than younger workers [15, 16]. However, other studies have found that workers find themselves spending more time preparing for e-learning, which entails a higher workload and the production of work of lesser quality, especially for those who do not have experience with e-learning [17]. Other challenges were reported related to technical issues, such as lack of access to the internet and lack of information technology (IT) support [18, 19]. Time management, stress, and anxiety when using e-learning are challenges and/or barriers for trainers and training coordinators [20]. In a recent study conducted in Saudi Arabia, Rajab et al. [21] identified the challenges that have been brought about by the COVID-19 pandemic as reported by medical students and faculty, including increased need for communication, use of novel technology tools, challenges in student assessment, technophobia, anxiety, or stress related to the pandemic, inexperience in online approaches, and time management.

Faculty members and students experience novel challenges in and barriers to online teaching and learning; however, no study has been conducted on these topics in the context of trainers and training coordinators working outside academic institutions. This study explores these challenges and barriers to e-learning experienced by trainers and training coordinators in the context of the Saudi MOH during the COVID-19 pandemic, as the sphere of online education in Saudi Arabia has received insufficient attention in general, as well as on this issue in particular.

## Methods

### Study design and setting

The questionnaire survey designed for this study had a descriptive and cross sectional design. It was administered in Saudi Arabia between April and June 2021.

### Sampling and participants

The study targeted Saudi trainers and training coordinators who were working at the time of the study in the Saudi MOH. The General Administration of Training and Scholarships of the Saudi MOH reported that there are about 800 trainers and training coordinators working in hospitals, primary health care, and training centers (including the general directorate of regions and clusters) in all 13 administrative regions of the country.

Adopting a 95% confidence level and 80% power for the study, a sample size of 262 was chosen: The final sample included 262 people, and convenience sampling was used to create the sample (For further information on sampling and participants, refer to the supplementary information file).

### Data collection

The survey was delivered in the form of an online survey using SurveyMonkey to gather the data. Using official emails of trainers and coordinators who were part of the study population, reminders were sent until the required sample size had been reached.

The questionnaire was adapted from data from two previously validated instruments and was used to collect required data. The questionnaire was first developed in English and then translated into Arabic. A bilingual panel of two healthcare experts and one externally qualified medical translator translated the English questionnaire into Arabic. Two English-speaking translators completed a back translation, and the original panel was consulted in case of any discrepancy.

The questionnaire was divided into four sections. The first section included information on the study and electronically solicited informed consent. Next, the second section presented questions on the participant’s sociodemographic characteristics, including gender, date of birth, position, and online teaching experience. Participants were divided according to generation, and classified as follows: 1946–1964 for Baby Boomers, 1965–1980 for Generation X, and 1981–1996 for Millennials or Generation Y [22]. Because the Hijri calendar is also used in Saudi Arabia, the date of birth was obtained in Gregorian dates to ensure comparability.

The third section used a survey adapted from Rajab et al. [21] and included questions related to communication, student assessment using technology tools, online experience, pandemic-related anxiety or stress, time management, and technophobia. The challenges to online learning and teaching were assessed on a four-point Likert scale: 1 = not a challenge, 2 = somewhat a challenge, 3 = a challenge, and 4 = a significant challenge. Lastly, the fourth section incorporated a survey that adapted Lloyd et al. [14]. This section included 22 variables to assess participants’ perceived barriers to online education as scored on a four-point Likert scale: 1 = not a barrier, 2 = somewhat a barrier, 3 = a barrier, and 4 = a significant barrier.

### Statistical analysis

All datasets were coded and analyzed using the Statistical Package for the Social Sciences version 23. Continuous variables were expressed as means and standard deviations, and categorical variables were expressed as the number of participants and percentages. One-way analysis of variance was adopted, taking e-learning level as a categorical independent variable for more than two groups, and the total challenges and barriers score as a quantitative normally distributed dependent variable; a t test was performed to obtain continuous variables. Cronbach’s alpha was used to assess the reliability of the questionnaire. Pearson’s correlation coefficient was used to clarify the correlation between the challenges and barriers. Univariate logistic regression was used to assess the factors associated with challenges and the barriers. The margin of error was 4.97±, and a p-value of 0.05 was considered statistically significant.

### Ethical considerations

The study protocol was reviewed and approved by the central institutional review board (IRB) of the Saudi MOH. The conduct of the study was guided by the ethical standards of the IRB of the Saudi MOH. Privacy and confidentiality were respected throughout the entire study. The research information and the rights of the participants were properly and adequately explained to the participants as they began the survey. All participants are anonymous, informed written consent was solicited from the participants online before the survey, and they were informed that their participation was voluntary.

## Results

Table 1 reports the demographics of the questionnaire participants, including gender, age group, and level of experience with e-learning among participants during the COVID-19 crisis. In all, the data from 262 participants were collected by the online survey and were included in the study; 139 of the participants (53.1%) were male. It is also evident that a large percentage of respondents were training coordinators, representing 53.8% of participants, while trainers represented 46.2% of participants. Most participants (64.9%) were members the age group born between 1981 and 1996 (Millennials or Generation Y). The level of e-learning experience among participants was that of an intermediate level (153, 58.4%), while nearly one-fourth of participants had an advanced level of experience (64, 24.4%).

**Table 1:** Demographic characteristics of the participants.

Demographic characteristics	Number of participants (N) = 262	Percentage (%)
**Gender**		
**Male**	139	53.1
**Female**	123	46.9
**Year of birth**		
**1946–1964**	10	3.8
**1965–1980**	82	31.3
**1981–1996**	170	64.9
**Position**		
**Trainer**	121	46.2
**Coordinator**	141	53.8
**E-learning level**		
**Beginner (little or no experience)**	45	17.2
**Intermediate**	153	58.4
**Advanced**	64	24.4

The most significant challenges reported as having occurred during the COVID-19 pandemic were the learning curve (adapting to unfamiliar technology) (22.1%), followed by experience in online teaching (20.2%), and assessment of trainees (19.5%). However, most participants (37.4%) categorized technophobia as not a challenge, which was followed by time management, mental health (stress, anxiety), and trainee evaluation of trainers/coordinators (29.8%, 28.2%, and 18.7%, respectively; Table 2).

**Table 2:** Challenges to e-learning experienced by trainers and coordinators in the Saudi MOH during the COVID-19.

Challenges	Not a challenge	Somewhat a challenge	A challenge	A significant challenge
**Communication, n (%)**	39 (14.9)	99 (37.8)	83 (31.7)	41 (15.6)
**Assessment of trainees, n (%)**	37 (14.1)	91 (34.7)	83 (31.7)	51 (19.5)
**Use of technology tools (access to hardware and software), n (%)**	41 (15.6)	108 (41.2)	68 (26.0)	45 (17.2)
**Experience in online teaching, n (%)**	45 (17.2)	98 (37.4)	66 (25.2)	53 (20.2)
**Mental health (stress, anxiety), n (%)**	74 (28.2)	118 (45.0)	46 (17.6)	24 (9.2)
**Learning curve (adapting to unfamiliar technology), n (%)**	26 (9.9)	110 (42.0)	68 (26.0)	58 (22.1)
**Trainee evaluation of trainers/coordinators, n (%)**	49 (18.7)	95 (36.3)	80 (30.5)	38 (14.5)
**Time management, n (%)**	78 (29.8)	111 (42.4)	48 (18.3)	25 (9.5)
**Technophobia, n (%)**	98 (37.4)	102 (38.9)	42 (16.0)	20 (7.6)

Table 3 shows the barriers to e-learning among participants. A lack of social interaction in the context of cheating/plagiarism had the highest percentage (46.9%). Frequent technological failure scored the next highest percentage (43.5%), and the third most significant barrier for participants was a lack of e-learning policies or standards (40.1%). By contrast, lack of course quality, personal anxiety/fear in the context of technology/online teaching, and the impersonal character of e-learning were ranked as being somewhat of a barrier by 43.1%, 41.6%, and 40.1% of participants, respectively. However, participants recognized factors, such as time commitment (34.0%), increased workload (27.5%), and lack of involvement in course decision-making (25.2%), as posing no impact on e-learning, thus rating those factors as nonbarriers (Table 3).

**Table 3:** Barriers to e-learning experienced by trainers and coordinators in the Saudi MOH during the COVID-19 pandemic.

Barriers	Not a barrier	Somewhat a barrier	A barrier	A significant barrier
**Lack of personal relationship with trainees, n (%)**	35 (13.4)	96 (36.6)	81 (30.9)	50 (19.1)
**Impersonal, n (%)**	66 (25.2)	105 (40.1)	51 (19.5)	40 (15.3)
**Lack of course quality, n (%)**	28 (10.7)	113 (43.1)	66 (25.2)	55 (21.0)
**Lack of visual cues from trainees, n (%)**	23 (8.8)	69 (26.3)	92 (35.1)	78 (29.8)
**Lack of social interaction among the class, n (%)**	14 (5.3)	62 (23.7)	97 (37.0)	89 (34.0)
**Lack of policies or standards for e-learning, n (%)**	17 (6.5)	61 (23.3)	79 (30.2)	105 (40.1)
**Lack of control over property rights, n (%)**	38 (14.5)	103 (39.3)	75 (28.6)	46 (17.6)
**Lack of involvement in course decision-making, n (%)**	66 (25.2)	104 (39.7)	59 (22.5)	33 (12.6)
**Online work not valued for promotion and tenure, n (%)**	75 (28.6)	91 (34.7)	56 (21.4)	40 (15.3)
**Inadequate trainer or coordinator training, n (%)**	34 (13.0)	53 (20.2)	95 (36.3)	80 (30.5)
**Inadequate technology support, n (%)**	6 (2.3)	49 (18.7)	106 (40.5)	101 (38.5)
**Frequent technology failure, n (%)**	3 (1.1)	47 (17.9)	98 (37.4)	114 (43.5)
**Rapidly changing software or delivery systems, n (%)**	31 (11.8)	80 (30.5)	78 (29.8)	73 (27.9)
**Increased workload, n (%)**	72 (27.5)	72 (27.5)	67 (25.6)	51 (19.5)
**Time commitment, n (%)**	89 (34.0)	93 (35.5)	53 (20.2)	27 (10.3)
**Inadequate time for grading and feedback, n (%)**	24 (9.2)	91 (34.7)	83 (31.7)	64 (24.4)
**Inadequate compensation for instruction, n (%)**	64 (24.4)	75 (28.6)	67 (25.6)	56 (21.4)
**Lack of social interaction in the context of cheating/plagiarism, n (%)**	14 (5.3)	38 (14.5)	87 (33.2)	123 (46.9)
**Lack of enrollment limits, n (%)**	38 (14.5)	74 (28.2)	75 (28.6)	75 (28.6)
**Lack of internet access for trainees, n (%)**	9 (3.4)	61 (23.3)	92 (35.1)	100 (38.2)
**Personal anxiety/fear in the context of technology/online teaching, n (%)**	72 (27.5)	109 (41.6)	45 (17.2)	36 (13.7)

**Fig 1:**
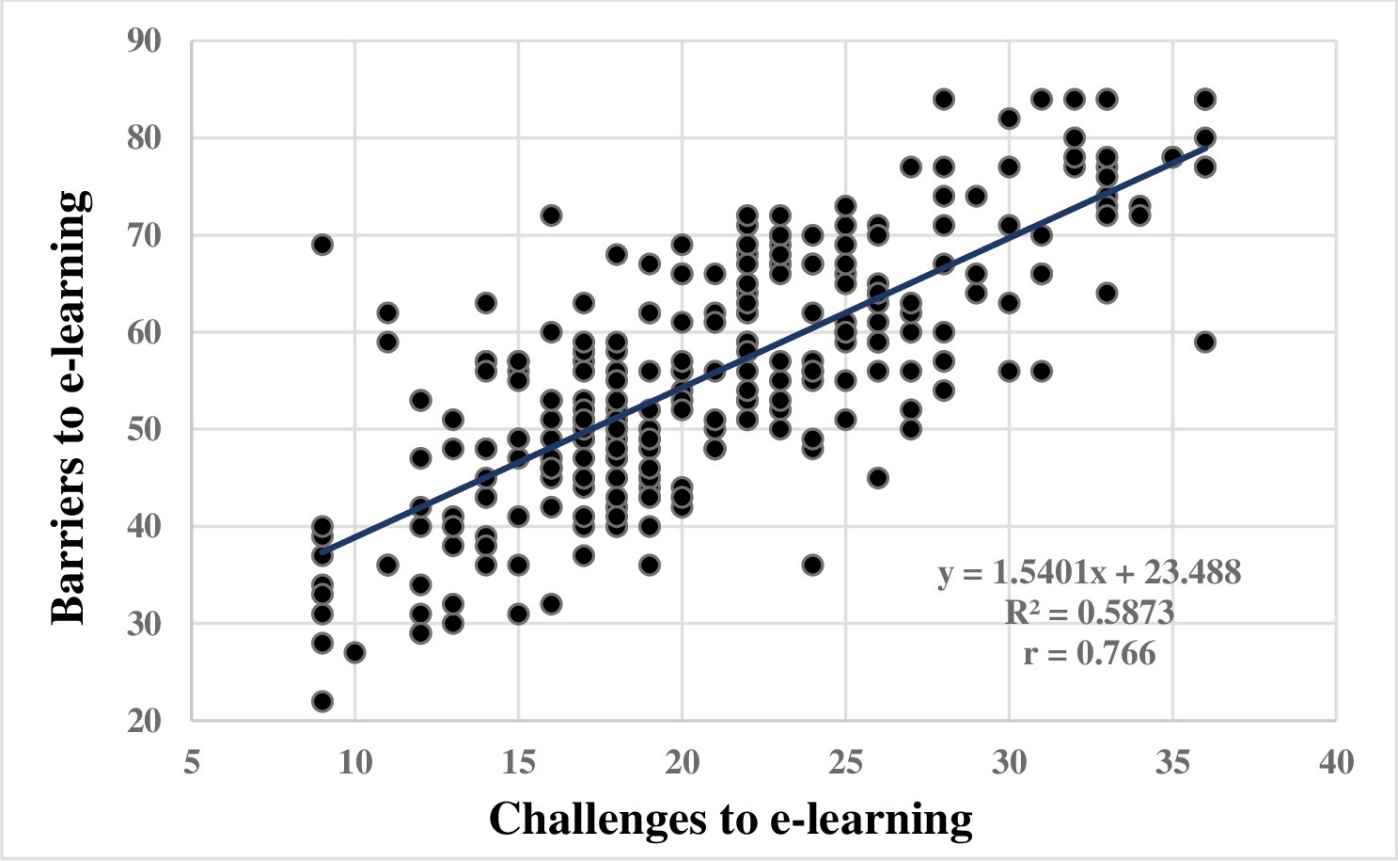
Pearson’s correlation coefficient between challenges and barriers, total scores.

There was a significant association between the challenges and barriers to e-learning experienced by trainers and training coordinators working in the MOH of Saudi Arabia, and a positive correlation was found between the two dimensions since r = 0.766 and p < 0.0001 (r is the Pearson correlation coefficient) (Fig 1).

As shown in Table 4, a nonsignificant correlation existed between male and female participants regarding challenges and barriers to e-learning in the MOH during the COVID-19 crisis, with p-values of 0.307 and 0.377, respectively. The level of experience with e-learning was significantly correlated, with p-values of <0.0001 and <0.0001, respectively.

**Table 4:** Challenges and barriers to e-learning in relation to participant data.

Challenges/barriers	Category	Mean	Std. Dev.	Median	Min.	Max.	P-value
**Challenges to e-learning**	Male	20.640	6.043	20.0	9.0	36.0	0.307
	Female	21.577	7.124	21.0	9.0	36.0	
**Barriers to e-learning**	Male	55.273	13.066	56.0	22.0	84.0	0.377
	Female	56.724	13.397	57.0	27.0	84.0	
**Challenges to e-learning**	Trainer	22.107	6.7895	22.0	9.0	36.0	0.043
	Coordinator	20.199	6.2795	20.0	9.0	36.0	
**Barriers to e-learning**	Trainer	57.851	13.108	56.0	22.0	84.0	0.051
	Coordinator	54.326	13.138	55.0	28.0	84.0	
**Challenges to e-learning**	Beginner	26.178	7.429	26.0	9.0	36.0	<0.0001
	Intermediate	20.758	5.509	21.0	9.0	33.0	
	Advanced	18.266	6.368	17.0	9.0	36.0	
**Barriers to e-learning**	Beginner	65.622	13.888	67.0	33.0	84.0	<0.0001
	Intermediate	55.804	11.605	56.0	22.0	84.0	
	Advanced	49.516	12.456	49.5	27.0	84.0	

Tables 5 and 6 show the univariate logistic regression for factors associated with challenges and barriers to e-learning as experienced by trainers and training coordinators in the Saudi MOH during the COVID-19 pandemic. The gender of the participants was not significantly associated with challenges and barriers to e-learning, showing p-values of 0.908 and 0.895, respectively. However, for beginners and intermediate participants, level of experience was significantly associated with challenges and barriers to e-learning, representing p-values of (0.001, 0.003) and (0.007, 0.004), respectively.

**Table 5:** Univariate logistic regression for factors associated with challenges to e-learning experienced by trainers and coordinators.

Demographic data		Odds ratio	95% CI		P-value
			Lower	Upper	
**Gender**	**Male**	0.97	0.577	1.631	0.908
	**Female** **	1			
**Position**	**Trainer**	1.135	0.674	1.913	0.634
	**Coordinator** **	1			
**E-learning level**	**Beginner**	8.516	2.977	24.361	< 0.001*
	**Intermediate**	2.476	1.359	4.511	0.003*
	**Advanced** **	1			

*Significant p-value

**Used as a reference

**Table 6:** Univariate logistic regression for factors associated with barriers to e-learning experienced by trainers and coordinators.

Demographic data		Odds ratio	95% CI		P-value
			Lower	Upper	
Gender	Male	0.954	0.475	1.917	0.895
	Female**	1			
Position	Trainer	3.657	1.602	8.347	0.002*
	Coordinator**	1			
E-learning level	Beginner	17.217	2.204	134.5	0.007*
	Intermediate	2.935	1.409	6.114	0.004*
	Advanced**	1			

*Significant p-value

**Used as a reference

## Discussion

E-learning was adopted in Saudi Arabia during the early 1990s and was expanded in the following decades as computer technology developed [23]. However, the cessation of educational activities in Saudi Arabia due to the ongoing COVID-19 pandemic has resulted in a massive unplanned shift from traditional learning to digital teaching and learning. This sudden transition without prior preparation has exposed some barriers and challenges to e-learning [24].

The challenges to e-learning most often identified by participants were the learning curve, experience in online teaching, assessment of trainees, and use of technological tools. These findings are consistent with the results found by Coman et al. [25], who analyzed the hierarchy of problems that arise together with changes in online learning in the context of COVID-19. They also highlighted technical problems as the most frequently reported factor, and educators’ lack of technical skills also decreased students’ motivation. The findings of this survey are aligned with those of Khalil et al. [24], who found that their participants encountered numerous challenges in adopting online learning. One of the most common barriers, aside from technical insufficiency and poor internet connectivity, was insufficient technical skill among educators. Khalil et al. [24] indicated that the efficiency of the use of technology in medical education depends on the faculty’s readiness and expertise in using the given technology in facilitating learning. The second issue was that most instructors had no experience delivering online lectures. According to students, time was wasted every day due to technical problems [24].

More than half of the participants in this study had a moderate level of experience in e-learning, while nearly one-quarter had an advanced level of experience. However, the trainers and coordinators faced challenges when using e-learning. These challenges may stem from a sudden transition to e-learning that provided insufficient time to adapt to the new ways, which made each trainer and coordinator depend on their background knowledge and skills in using digital technology. A unified training program and supplementary workshops may be necessary to train how to effectively use digital platforms for e-learning.

Additionally, communication challenges were faced by the trainers and training coordinators who participated in the study. Communication was mentioned by several studies as a challenge to e-learning. A national survey of 3670 medical students from 54 schools in the Philippines indicated that communication channels required improvement in the context of e-learning. Students commented on the lack of appropriate action from school administrators in response to student feedback on the conduct of e-learning [26]. Padhi et al. [27] reported that 91.7% of educators indicated that communication skills were the most essential skill for teaching effectively in the context of e-learning, followed by technological skills.

However, in this study, technophobia and mental health (stress, anxiety) were not considered by participants as a challenge to e-learning. These results were consistent with those of a previous systemic literature review completed by Regmi and Jones [28], who examined barriers to e-learning. That review shows that there are internal factors related to barriers to e-learning, including poor engagement and motivation, limited flexibility, and high levels of anxiety and stress. Moreover, time management was also not identified as a challenge in this study, which supports the results of Zalat et al. [29].

These findings indicate participants’ readiness and acceptance toward e-learning, although they face challenges when using e-learning. This is due to the current challenges posed by the pandemic and the adaptation to digital culture in daily life, including the training and education contexts.

However, the findings in this study indicated that for most respondents, the lack of social interaction in the context of cheating/plagiarism and the impersonal character of e-learning were significant barriers for them. Similarly, Bylieva et al. [30] showed that the levels of academic dishonesty had grown and extended to e-learning. They also reported that the principles of academic ethics that were violated include scientific integrity, the inadmissibility of plagiarism, and compliance with the rules in the assessment of students’ knowledge and achievements [30]. Moreover, the lack of policies or standards for e-learning was a significant barrier to e-learning for trainers and coordinators, together with a lack of course quality. Likewise, a national survey conducted among medical students in the Philippines indicated that these students faced several interrelated barriers as they adapted to online learning. The students cited a lack of guidelines and policies, haphazard class schedules, low-quality teaching materials, ineffective teaching strategies, and excessive class requirements as barriers [27]. Additionally, the unavailability of policy documents raised concerns regarding the attainment of specific objectives in e-learning and continued training during the pandemic, which caused low-quality remote learning [31–33].

However, as has been shown in the results of this study, many previous studies have indicated frequent technological failures as a barrier to e-learning [25, 27, 29], and they all highlighted the major problems faced by educators while conducting online sessions as a result of network issues.

The Saudi MOH has succeeded in implementing and maintaining an optimized strategy to minimize the spread of COVID-19 using various digital health technology platforms to ensure social distance is maintained. One such platform is Microsoft Teams, which is used to conduct meetings, training sessions, and workshops among the Saudi MOH employees. However, there is strong financial and logistic support from the Saudi MOH for all digital health platforms to ensure access. IT support for Microsoft Teams only require that all MOH employees have a Microsoft Teams account. Trainers and coordinators, however, need clear policies and guidelines for using Microsoft Teams in e-learning. Policymakers, IT support, and other stakeholders may need to establish clear policy guidelines on the use of digital platforms in e-learning, which cover aspects such as using and familiarizing oneself with icons, preventing cheating, and effectively communicating with trainees. Furthermore, IT support roles could be available when technical issues arise during training sessions, meetings, or workshops. Detailed tutorial videos focusing on how to use digital platforms could be provided.

Nonetheless, time commitment and increased workload were not considered to be barriers by the respondents. This result is in line with the findings of Coman et al. [25], who found that, unlike face-to-face learning, e-learning has become popular mainly because of its flexibility in delivering education and providing access to content and resources. Thus, e-learning has great importance because of its ability to improve the quality of instruction, offering the possibility of personalizing and adapting courses to the needs of learners.

Moreover, this study showed that the gender of the participants was not significantly associated with challenges and barriers to e-learning. This result agrees with the findings of Gamdi and Samarji [34], who investigated the challenges of adopting e-learning at higher-level Saudi universities. They concluded that there is no consensus that the perceived barriers to e-learning are gender-specific. It was also found that female faculty members perceived fewer e-learning barriers than their male counterparts. By contrast, Zalat et al. [29] reported that women showed a greater preference for e-learning than men.

The different levels of experience between beginners and intermediate participants were also significantly associated with challenges and barriers to e-learning. This finding is in agreement with the work of Shahmoradi et al. [19], who stated that students with greater online experience have a greater level of satisfaction with online learning, and these authors found relationships among skill, experience, and cultural challenges in using e-learning systems.

### Limitations

This study had a few limitations. Its findings cannot be generalized because, as a cross sectional study, the respondents’ perceptions may change over time. Therefore, further longitudinal study and regular evaluations are required to obtain an improved understanding of challenges and barriers to the adoption of e-learning systems in the Saudi MOH. Another limitation that should be noted is that the study focused on identifying challenges and barriers to online learning and their relative importance from the point of view of trainers and training coordinators and therefore did not delve into the participants’ expectations, motivations, and perceptions with respect to e-learning, which also influence their overall learning experience.

### Conclusions

This study identified the challenges and barriers encountered in the adoption of e-learning by trainers and training coordinators in the Saudi MOH. Communication, assessment of trainees, experience with online teaching, adaptation to unfamiliar technology, and the ability to use different technological tools were challenges for participants. The barriers to e-learning included but were not limited to a lack of policy and frequent technological failure. Most of the challenges and barriers found by this study were related to lack of proper training, policies, and guidelines that pertain to e-learning. The pandemic period may be an opportunity to make positive changes in relation to e-learning, by establishing clear policies and guidelines to use e-learning, unified training program and workshops regardless of trainers’ and coordinators’ experiences in e-learning, and continuous IT support, especially when setting up a new digital platform. The findings of this study may give policymakers and developers of digital transformation a view of the current situation to improve e-learning as a tool of learning among trainers and coordinators.

## Supporting information

S1 File Sampling and participants. (DOCX)

## List of abbreviations

E-learning: Electronic Learning
IT: Information Technology
MOH: Ministry of Health
WHO: World Health Organization
