# Supplementary material for: Challenges and barriers to e-learning experienced by trainers and training coordinators in the Ministry of Health in Saudi Arabia during the COVID-19 crisis
Source: PLoS One. 2022 Oct 17;17(10):e0274816. doi: 10.1371/journal.pone.0274816 (PMC9576076; doi:10.1371/journal.pone.0274816)
Supplement: S1 File — (DOCX) [file pone.0274816.s001.docx]

**Supporting information**

**S1 File. Sampling and participants:**

- Several equations were used to determine the sample size, one of which was the OpenEPI website, which consistently generated a sample count of 262 participants. The margin of error observed in the study was 4.97± and the confidence level was 95%.
- In total, 578 emails were sent to the participants from the Saudi Ministry of Health (MOH)’s General Administration of Training and Scholarships after obtaining Institutional Review Board (IRB) approval. Only 578 emails were sent because the list of emails was not updated. Post that, 80 emails were eliminated because they were inaccurate. This reduced the total number of participants to 498. I conducted weekly follow-ups until I received 262 completed questionnaires, after which I stopped with the follow-ups.
- Responses were sought from trainers and coordinators who were included in the study through official emails, and weekly reminders were sent until the required sample was completed.
- Total participants who responded ÷ total participants who were sent the questionnaire × 100 =
- 262 ÷ 498 × 100 = 45.3%.
